# Let-7f-5p Modulates Lipid Metabolism by Targeting Sterol Regulatory Element-Binding Protein 2 in Response to PRRSV Infection

**DOI:** 10.3390/vetsci11090392

**Published:** 2024-08-26

**Authors:** Dongfeng Jiang, Liyu Yang, Xiangge Meng, Qiuliang Xu, Xiang Zhou, Bang Liu

**Affiliations:** 1 Key Laboratory of Agricultural Animal Genetics, Breeding and Reproduction of Ministry of Education, Huazhong Agricultural University, Wuhan 430070, China; jdf13526758315@163.com (D.J.); mengxg@webmail.hzau.edu.cn (X.M.); 2 College of Animal Science and Technology, Henan University of Animal Husbandry and Economy, Zhengzhou 450046, China; 18738133860@163.com (L.Y.); 15136251005@163.com (Q.X.); 3 Henan Institute of Pig Biotech Breeding, Zhengzhou 450046, China; 4 The Engineering Technology Research Center of Hubei Province Local Pig Breed Improvement, Huazhong Agricultural University, Wuhan 430070, China; 5 Hubei Hongshan Laboratory, Wuhan 430070, China

**Keywords:** let-7f-5p, SREBP2, PRRSV, lipogenesis, RNA-seq

## Abstract

**Simple Summary:**

Porcine reproductive and respiratory syndrome (PRRS) caused by PRRS virus (PRRSV) leads to considerable economic losses for the global pig industry. Investigating PRRSV–host interactions is important for understanding PRRSV pathogenesis and developing antiviral strategies. This study focuses on the post-transcriptional regulation of let-7f-5p in response to PPRSV infection. Lipids play a key role in the replication cycle of PRRSV. We found that let-7f-5p directly targets the master lipid metabolism regulator SREBP2 and influences the expression of lipogenesis genes, which provide a novel target for PRRSV antiviral therapy.

**Abstract:**

Porcine reproductive and respiratory syndrome (PRRS) has caused substantial damage to the pig industry. MicroRNAs (miRNAs) were found to play crucial roles in modulating the pathogenesis of PRRS virus (PRRSV). In the present study, we revealed that PRRSV induced let-7f-5p to influence lipid metabolism to regulate PRRSV pathogenesis. A transcriptome analysis of PRRSV-infected PK15^CD163^ cells transfected with let-7f-5p mimics or negative control (NC) generated 1718 differentially expressed genes, which were primarily associated with lipid metabolism processes. Furthermore, the master regulator of lipogenesis SREBP2 was found to be directly targeted by let-7f-5p using a dual-luciferase reporter system and Western blotting. The findings demonstrate that let-7f-5p modulates lipogenesis by targeting SREBP2, providing novel insights into miRNA-mediated PRRSV pathogenesis and offering a potential antiviral therapeutic target.

## 1. Introduction

Porcine reproductive and respiratory syndrome (PRRS) is a highly infectious disease that severely affects swine health and causes significant financial losses for the global pig industry [1]. Clinical symptoms of PRRS are characterized by late-term reproductive failure in sows and severe pneumonia in piglets. The PRRS virus (PRRSV) genome is a single-stranded RNA of approximately 15 kb, with 11 open reading frames (ORFs) [2]. PRRSV strains are divided into two principal genotypes: type 1 PRRSV (European) and type 2 PRRSV (North American), distinguished by their genomic diversity [3]. PRRSV specifically infects cells of the monocyte–macrophage lineage and prefers to target primary pulmonary alveolar macrophages (PAMs) in the lung and macrophages in other tissues [4,5]. PRRSV infection is associated with immunosuppression and a robust inflammatory response, leading to thymocyte apoptosis in piglets and subsequent thymus atrophy [6,7]. The ongoing emergence of recombinant mutant strains worldwide presents a significant challenge in effectively preventing and controlling PRRSV. Therefore, investigating the mechanisms underlying PRRSV–host interactions is crucial for developing antiviral strategies.

MiRNAs play a crucial role in controlling the regulation of infection and replication of numerous viruses, including PRRSV [8], porcine delta coronavirus (PDCoV) [9], transmissible gastroenteritis virus (TGEV) [10] and porcine epidemic diarrhea virus (PEDV) [11]. MiRNAs can directly target and regulate PRRSV genome replication. For instance, miRNA-122 [12], miR-23 [13], miR-150 [14] and miR-218 [15] have been identified as being able to target viral RNA and inhibit PRRSV production. In addition, miRNAs can influence the host’s immune system to regulate PRRSV replication. MiR-125b restricts PRRSV replication by blocking NF-κB pathway activation [16]. PRRSV triggers miR-218 to target SOCS3 and suppress SOCS3 expression, which inhibits host IFN signaling and increases PRRSV replication [15]. Let-7 is recognized as a crucial regulator in development and cancer, and the porcine let-7 family comprises eight miRNAs with similar sequences [17,18]. The let-7 family modulates various viral infections by affecting both innate and adaptive immune response pathways [19]. The let-7 family has been shown to inhibit PRRSV replication by targeting the 3′ UTRs of the PRRSV genome and IL-6, which play a crucial role in both PRRSV replication and lung injury [20]. The interaction between MYH9 and the GP5 protein of PRRSV enhances PRRSV replication [21]. Let-7f-5p inhibits MYH9 expression by binding to the 3′UTR of MYH9 mRNA and restricts PRRSV replication [22].

The let-7 family has been reported to regulate glucose and lipid metabolism in different cells [23,24]. Lipids are essential for the replication of both enveloped and non-enveloped viruses [25]. Viruses can induce the accumulation of triglyceride (TG) in infected cells and facilitate viral replication [26]. PRRSV infection induces the accumulation of intracellular TG, which serves as a vital resource for PRRSV replication [27]. Reducing the accumulation of TG and lipogenesis by silencing SMPDL3B effectively inhibits PRRSV infection [28]. Sterol regulatory element-binding protein 2 (SREBP2) is a transcription factor that regulates cholesterol biosynthesis and influences cellular lipid levels [29,30]. SREBP2 has been reported to regulate lipid accumulation in the white adipose tissue of animals through let-7a-5p/Srebf2 axes [31]. In the present study, we have demonstrated that the up-regulation of let-7f-5p inhibited lipogenesis in PRRSV-infected cells through directly targeting the 3′UTR of *SREBP2* mRNA. These findings indicate that the miRNA-mediated regulation of lipid metabolism plays a crucial role in PRRSV pathogenesis and suggest a potential target for the future control of PRRSV infection.

## 2. Materials and Methods

### 2.1. Cells and Viruses

PK15 and PK15^CD163^ cells were maintained in Dulbecco’s Modified Eagle’s Medium (DMEM) (Gibco, South Logan, USA) containing 10% Fetal Bovine Serum (FBS) (Gibco, South Logan, UT, USA) and incubated at 37 °C with 5% CO_2_. The PRRSV strain WUH3 was kindly shared by Dr. Xiao Shaobo from Huazhong Agricultural University.

### 2.2. miRNAs, Plasmids and Target Prediction

The let-7f-5p mimics, negative control (NC), let-7f-5p inhibitor and inhibitor negative control were synthesized by GenePharma (Shanghai, China). The sequences are shown in Supplementary Table S1. The sequence of the partial 3′UTR of *SREBP2* was amplified using PCR with Pyrobest DNA Polymerase (Takara, Beijing, China) and then cloned into the psi-CHECK2 vector using the high-fidelity enzymes NotI-HF and XhoI (NEB, Ipswitch, MA, USA) to create a dual-luciferase vector named psi-CHECK2-SREBP2-3′UTR. The PCR primers are shown in Supplementary Table S1. The prediction of target genes for let-7f-5p was performed using the TargetScanHuman v8.0 software (https://www.targetscan.org/vert_80/, accessed on 1 July 2024).

### 2.3. Triglyceride (TG) Level Determination

PK15 cells were seeded into 6-well plates and transfected with 50 nM of let-7f-5p mimics, let-7f-5p inhibitor and NC. After 36 h of incubation, the cells were lysed and heated at 70 °C for 10 min, then centrifuged to collect the supernatants. Triglyceride (TG) levels were then measured using an enzyme-linked reader (PerkinElmer, Singapore) with the Triglyceride GPO-POD Assay Kit (Applygen, Beijing, China).

### 2.4. Transcriptomic Analysis

PK15^CD163^ cells were seeded into 6-well plates and transfected with 50 nM of let-7f-5p mimics and NC. Following transfection, the cells were infected with PRRSV at a multiplicity of infection (MOI) of 1 for 1 h. The infection medium was then replaced with maintenance medium containing 2% FBS. The cells were then incubated for 24 h at 37 °C with 5% CO₂, after which the PRRSV-infected PK15^CD163^ cells were harvested for RNA-seq analysis. Total RNA was extracted using RNAI-Plus (Takara, Tokyo, Japan). RNA-seq library construction and sequencing were performed using the Illumina platform at Wuhan SeqHealth Tech Co., Ltd. (Wuhan, China). Subsequently, the raw data were filtered, and the clean data were aligned with the reference genome of Sus scrofa 11.1. The differentially expressed genes (DEGs) between groups were screened by Fold Changes > 1.5 and FDR < 0.05 based on DESeq2. Metascape was applied to conduct functional and pathway enrichment for the DEGs [32].

### 2.5. Quantitative Real-Time PCR

The total RNA used in the RNA-seq analysis was performed to conduct quantitative Real-Time PCR (qRT-PCR). The PrimeScript™RT reagent kit (TAKARA, Tokyo, Japan) was used to revise the total RNA used in Section 2.4 to generate cDNA. A qRT-PCR analysis of cDNA was performed using the TB Green^®^ Premix Ex Taq™ (TAKARA, Tokyo, Japan) on a Bio-Rad CFX384 system (Bio-Rad, Richmond, VA, USA). PCR primers are shown in Supplementary Table S1.

### 2.6. Western Blot

PK-15 cells were seeded into a 6-well plate and transfected with 50 nM of let-7f-5p mimics and NC. After 36 h of transfection, total proteins were extracted from the cells using RIPA lysis buffer (Beyotime, Shanghai, China), supplemented with PMSF (Beyotime, Shanghai, China) and phosphatase inhibitor cocktail I (MedChemExpress, Shanghai, China). Total proteins were separated by SDS-PAGE and transferred to PVDF membranes (Millipore, Billerica, MA, USA). The membranes were subsequently blocked with 5% nonfat milk for 2.5 h at room temperature and then incubated with anti-SREBP2 primary antibody (Proteintech, Rosemont, IL, USA, 28212-1-AP, 1:1000) overnight at 4 °C. Following three washes with TBST, the PVDF membranes were incubated at room temperature for 2 h with the secondary antibody (ABclonal, Woburn, MA, USA, 1:5000). The immunoblots were visualized using ImageQuant LAS4000 mini (GE Healthcare Life Sciences, Piscataway, NJ, USA).

### 2.7. Luciferase Reporter Assay

PK-15 cells were seeded into a 24-well plate. A total of 20 nM of let-7f-5p mimics, let-7f-5p inhibitor or NC was co-transfected with 500 ng of WT-SREBP2-3’UTR plasmids using Lipofectamine™ 2000 (Invitrogen™, Carlsbad, CA, USA). Luciferase activity was detected in each group using an automated microplate reader (PerkinElmer, Singapore) following the instructions provided with the Dual-Luciferase^®^ Reporter Assay System (Promega, Madison, WI, USA).

### 2.8. Statistical Analysis

All data had at least three biological replicates, and the results were analyzed using GraphPad Prism 7. Student’s *t*-test was used to examine the differences between the experimental and control groups, and they were presented as the mean ± SD. *p*-value < 0.001, *p*-value < 0.01 and *p*-value < 0.05 were considered as statistically significant.

### 2.9. Data Available

Raw sequencing data with accession number PRJNA1061237 are available in the NCBI Sequence Read Archive (SRA).

## 3. Results

### 3.1. Let-7f-5p Restricts PRRSV Replication and Regulates Lipid Metabolism

**Figure 1:**
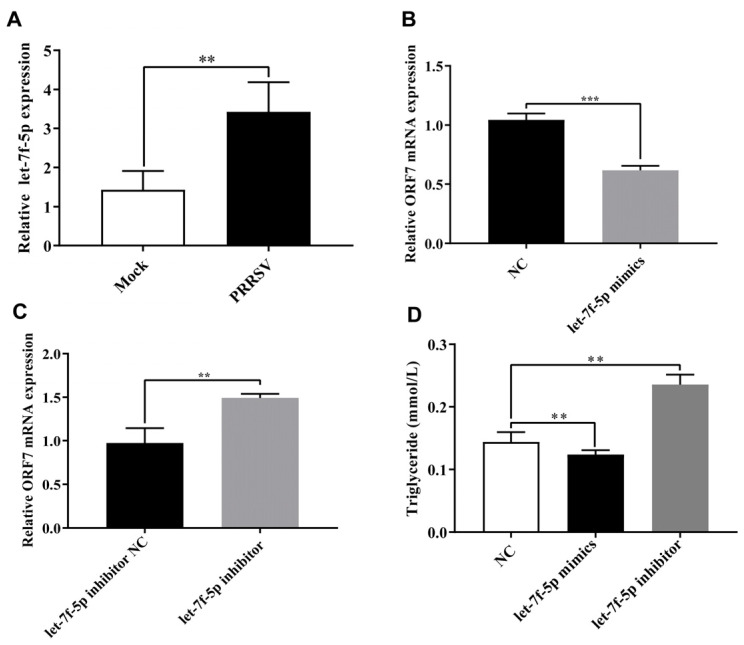
Let-7f-5p restricts PRRSV replication and regulates triglyceride production. (**A**) The relative expression of let-7f-5p during PRRSV infection. (**B**) The effect of let-7f-5p mimics on PRRSV replication. (**C**) The effect of the let-7f-5p inhibitor on PRRSV replication. (**D**) The effect of the let-7f-5p mimics or inhibitor on triglyceride production. (** *p* < 0.01, *** *p* < 0.001).

Compared with the negative control, let-7f-5p showed approximately a 2-fold increase in PRRSV-infected PK-15^CD163^ cells (Figure 1A). To assess the impact of let-7f-5p on PRRSV replication, the let-7f-5p mimics and let-7f-5p inhibitor were transfected into PRRSV-infected PK-15^CD163^ cells to analyze the expression of PRRSV *ORF7* mRNA. The addition of let-7f-5p mimics led to a significant reduction in the expression of PRRSV *ORF7* mRNA (*p* < 0.001) (Figure 1B). In contrast, treatment with the let-7f-5p inhibitor significantly increased the expression of PRRSV ORF7 mRNA (*p* < 0.01) (Figure 1C). To verify the effect of let-7f-5p on regulating lipid metabolism, the let-7f-5p mimics and let-7f-5p inhibitor were transfected into PK-15 cells to test the TG level. With the addition of let-7f-5p mimics, the TG production of PK-15 cells was significantly decreased (*p* < 0.01), whereas when treated with the let-7f-5p inhibitor, the TG level was significantly increased (*p* < 0.01) (Figure 1D), which indicated that let-7f-5p was closely related to lipid metabolism.

### 3.2. Let-7f-5p Altered Lipogenesis Pathway in PRRSV-Infected PK-15^CD163^ Cells

**Figure 2:**
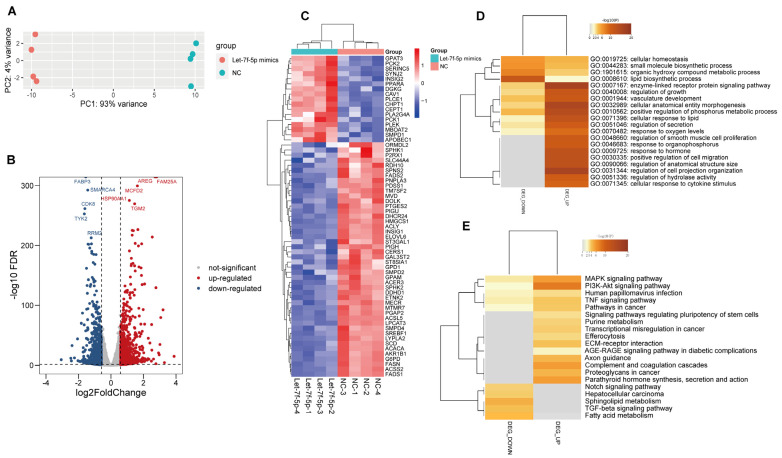
Let7f-5p altered the transcriptomic profile in PRRSV-infected PK-15^CD163^ cells. (**A**) The principal component analysis (PCA). (**B**) A volcano map of differentially expressed genes (DEGs). (**C**) A heatmap of DEGs related to lipid metabolism. (**D**) GO analysis. (**E**) KEGG pathway analysis.

To investigate the signaling pathways involved in let-7f-5p-mediated gene regulation in response to PRRSV infection, RNA-seq was performed on PRRSV-infected PK-15^CD163^ cells that were transfected with let-7f-5p mimics or the negative control (NC). PCA revealed that the let-7f-5p mimic-treated group was clearly separated from the NC group, indicating a significant difference in gene expression between the two groups (Figure 2A). In addition, a total of 1718 genes were significantly altered, with 896 genes being up-regulated and 822 genes down-regulated. The differently expressed genes (DEGs) included a lot of genes involved in lipid metabolism, including *FASN*, *FADS2*, *ELOVL6*, *ACLY*, *SREBPF*, *INSIG1* and others (Figure 2B,C). The Gene Ontology (GO) analysis revealed that the down-regulated differentially expressed genes (DEGs) are the most enriched in lipid biosynthetic processes (Figure 2D). Additionally, KEGG analysis showed that these down-regulated DEGs are primarily associated with the fatty acid metabolism pathway (Figure 2E). 

### 3.3. qRT-PCR Verified That Let-7f-5p Targeted Lipid Metabolism Pathway

**Figure 3:**
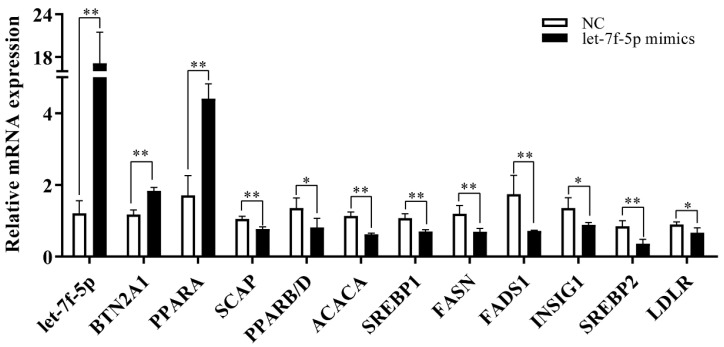
qRT-PCR analysis of expression of lipid metabolism genes in PRRSV-infected PK-15^CD163^ cells transfected with let-7f-5p or NC (* *p* < 0.05, ** *p* < 0.01).

To further investigate the impact of let-7f-5p on lipid metabolism in PRRSV-infected PK-15^CD163^ cells, the mRNA expression levels of several key genes involved in lipid metabolism were assessed using qRT-PCR. The results showed that the mRNAs of *BTN2A1* and *PPARA* were significantly up-regulated with the treatment of let-7f-5p in PK-15^CD163^ cells compared to the controls. However, the transcriptional levels of *SCAP*, *PPARB/D*, *ACACA*, *SREBP1*, *FASN*, *FADS1*, *INSIG1*, *SREBP2* and *LDLR* were significantly down-regulated (Figure 3).

### 3.4. Let-7f-5p Directly Targets SREBP2 to Modulate Lipid Metabolism

**Figure 4:**
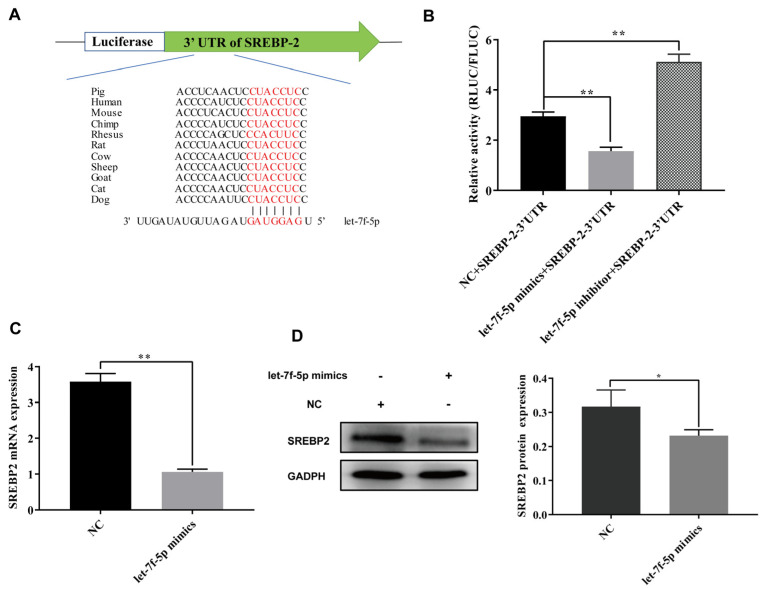
Let-7f-5p targeted mRNA of *SREBP2*. (**A**) Bioinformatical predicted target sequences of let-7f-5p to 3′UTR of SREBP2 from multiple species. (**B**) Luciferase activity of SREBP2-3’UTR from PK-15 cells transfected with let-7f-5p mimic, NC or let-7f-5p inhibitor. (**C**) mRNA expression of SREBP2 from PK-15 cells transfected with let-7f-5p mimic or NC. (**D**) Protein expression of SREBP2 from PK-15 cells transfected with let-7f-5p mimic or NC. (* *p* < 0.05, ** *p* < 0.01).

To explore the potential molecular mechanism of let-7f-5p in regulating host lipid metabolism in response to PRRSV infection, we predicted the target genes of let-7f-5p using TargetScan software 7.2. The results showed that the putative binding sites between *SREBP2* and let-7f-5p were highly conversed among different species, including pigs, mice, humans, chimps, sheep, cats and dogs (Figure 4A). The luciferase reporter assay was used to validate whether *SREBP2* is a direct target of let-7f-5p. The results showed a significant reduction in the RLUC/FLUC ratio in PK-15 cells treated with let-7f-5p mimics compared to the negative control (NC) group. In contrast, the addition of the let-7f-5p inhibitor led to a significant increase in the RLUC/FLUC ratio (*p* < 0.01) (Figure 4B), suggesting that let-7f-5p could target the 3’UTR of *SREBP2* mRNA. Furthermore, the expression level of SREBP2 showed a significant down-regulation in PK-15 cells treated with let-7f-5p mimics compared to NC, which was consistent with the results obtained from quantitative PCR and Western blot verification (Figure 4C,D). 

## 4. Discussion

Lipid metabolism and TG play a crucial role in the replication of various viruses [25]. To achieve rapid and extensive viral replication, viruses utilize cellular lipid signaling, synthesis and metabolic pathways to create favorable conditions for replication [33,34,35]. Previous studies have shown that HCV can manipulate the lipid metabolism of host cells to produce lipid droplets (LDs) rich in phospholipid layers and surface proteins, which contribute to viral assembly and release [36]. Lipid accumulation is promoted by PRRSV infection, and inhibiting LD accumulation leads to a significant reduction in PRRSV replication [27]. A recent study has reported that YY1 reprograms intracellular LD synthesis by altering the expression of FASN and PPARγ, thereby regulating PRRSV replication [37]. Epigallocatechin gallate (EGCG), extracted from green tea, restricts PRRSV replication and assembly by disrupting lipid metabolism [38]. The knockout of SMPDL3B results in the accumulation of intracellular LDs, limiting PRRSV attachment, entry, replication and secretion [28]. Through the modulation of the NF-κB signaling pathway, PRRSV enhances LD accumulation and promotes viral replication [27]. In the present study, we found that the up-regulation of let-7f-5p restricted TG production. In addition, the expression of lipid metabolism-related genes showed significant changes at the mRNA level when let-7f-5p was overexpressed in PRRSV-infected PK-15^CD163^ cells. The results of the present study showed that let-7f-5p could regulate host lipid metabolism to affect PRRSV infection. Furthermore, PK15 cells represent a well-established porcine kidney cell line commonly used in swine-related studies. PK-15^CD163^ cells are PK-15 cells modified to stably express CD163 and are specifically used in PRRSV infection experiments [39]. CD163 is primarily known as a scavenger receptor highly expressed on macrophages, particularly involved in the clearance of hemoglobin–haptoglobin complexes [40]. There is no well-established direct relationship between CD163 and lipid metabolism, which needs to be investigated in the future.

The accumulation of lipids in cells involves a series of enzyme-catalyzed reactions and is regulated by a variety of molecules. SREBPs mainly including SREBP1 and SREBP2 are a family of master transcription factors involved in the biosynthesis of endogenous phospholipids, TG, cholesterol and fatty acids (FAs), which are essential for maintaining lipid homeostasis [41]. SREBP2 activates the expression of genes involved in cholesterol biosynthesis by binding to sterol regulatory elements as well as the promoters and enhancers of genes related to lipid synthesis. The up-regulation of *PPARA* caused by let-7f-5p in cells can activate lipid oxidation and limit TG accumulation [42]. In addition, the down-regulation of lipid synthesis genes caused by let-7f-5p including *FASN*, *ACACA*, *SREBP1* and *SREBP2* can repress lipogenesis. SREBP2-dependent lipogenesis enhances Zika virus infection in dendritic cells [43]. AM580 compounds directly bind SREBP2 to regulate lipogenesis and restrict various RNA viruses including influenza A virus, SARS-Cov and Zika virus [44]. In this study, we demonstrated that let-7f-5p directly targets the 3′ UTR of the *SREBP2* gene to regulate lipid metabolism in response to PRRSV, which provides the research community with clues for future studies on the molecular mechanisms of PRRSV-induced lipid metabolism. This finding reveals a novel mechanism for PRRSV replication and new approaches for preventing and controlling PRRSV infection. 

## 5. Conclusions

The results from the present study showed that the up-regulation of let-7f-5p could modulate lipid metabolism in response to PRRSV infection. The underlying mechanism is that let-7f-5p directly targets SREBP2, which alters the expression of a wide range of lipid metabolic regulatory genes. Our data provide new research perspectives into the role of let-7f-5p in regulating PRRSV pathogenesis and lay a theoretical foundation for exploring new strategies to prevent PRRS prevalence.

## Data Availability

The original contributions presented in the study are included in the article/Supplementary Material, further inquiries can be directed to the corresponding author/s. Data are contained within the article.

## Supplementary Materials

The following supporting information can be downloaded at https://www.mdpi.com/article/10.3390/vetsci11090392/s1, Table S1: Sequence information.
