# Supplementary material for: Let-7f-5p Modulates Lipid Metabolism by Targeting Sterol Regulatory Element-Binding Protein 2 in Response to PRRSV Infection
Source: Vet Sci. 2024 Aug 26;11(9):392. doi: 10.3390/vetsci11090392 (PMC11435751; doi:10.3390/vetsci11090392)
Supplement: Supplementary file 1 [file vetsci-11-00392-s001.zip › vetsci-3106539-supplementary.pdf]

Supplemental Table S1. Primers used in PCR

Primers	Primer sequence (5'-3')
SUS-*GAPDH*-F	CGTCCCTGAGACACGATGGT
SUS-*GAPDH*-R	GCCTTGACTGTGCCGTGGAAC
ORF7-F	CATCGCCCAACAAAACCAG
ORF7-R	CGTCGGCAAACTAAACTCCA
miR-let-7f-5p-RT	CTCAACTGGTGTCGTGGAGTCGGCAATTCAGTTGAGAACTATAC
miR-let-7f-5p–F	CTGGTAGGTGAGGTAGTAGATT
miR-let-7f-5p -R	TCAACTGGTGTCGTGGAG
U6-94bp-F	CTCGCTTCGGCAGCACA
U6-94bp-R	AACGCTTCACGAATTTGCGT
BTN2A1-F	TTCCTTTACTCCCAGCAC
BTN2A1-R	CACATGATCTTTCCCTAGTTCCTTA
SREBP-1-F	AGCGGACGGCTCACAATG
SREBP-1-R	CGCAAGACGGCGGATTTA
SREBP-2-F	TGTGGAGCAGCCTCAATGTC
SREBP-2-R	TTTTGCCAGAGCACTGTCCG
INSIG1-F	TGTCGTGGGCTTGCTCTA
INSIG1-R	GCACTGGCGTGGTTGATG
SCAP-F	GCGGTGAGATTTTCCCCTAC
SCAP-R	GCCAATGAGGATGATGCC
PPARA-F	CAGCGTGGCACTGAACATC
PPARA-R	CTCCGATCACATTTGTCATAGAC
PPARB/D-F	TGGCTGGGCTGACGGCAAAC
PPARB/D-R	TCGATGTCGTGGATCACAAAGG
ACACA-F	GAAAATCCACAATGCCAACCC
ACACA-R	ACACCGTCTTCCTCCGTCAG
FADS1-F	TGAACCGACTAAGAATAAAGAG
FADS1-R	CCGAAGAGCCAAAGAATG
FASN-F	GCTTGTCCTGGGAAGAGTGTAA
FASN-R	GCAGGAACTCGGACATAGCG
LDLR-F	GACGAGGAGAACTGCGATGT
LDLR-R	GGCACTCATAGCCGATCTTG
psiCHECK-2-WT-SREBP-2 -3'UTR-F	CCGCTCGAGGGTATGGCTTCTCAGCTCCC
psiCHECK-2- WT-SREBP-2 -3'UTR-R	ATAAGAATGCGGCCGCCCAGGAGAAATGGCCTC CAG
psiCHECK-2-MUT-SREBP-2 -3'UTR-F	CCGCTCGAGACCTCAACTCCTAAAAACATTGTCT ATTT
psiCHECK-2-MUT -SREBP-2 -3'UTR-R	ATAAGAATGCGGCCGCCCAGGAGAAATGGCCTC CAG
