# Supplementary figures and images for: Let-7f-5p Modulates Lipid Metabolism by Targeting Sterol Regulatory Element-Binding Protein 2 in Response to PRRSV Infection
Source: Vet Sci. 2024 Aug 26;11(9):392. doi: 10.3390/vetsci11090392 (PMC11435751; doi:10.3390/vetsci11090392)

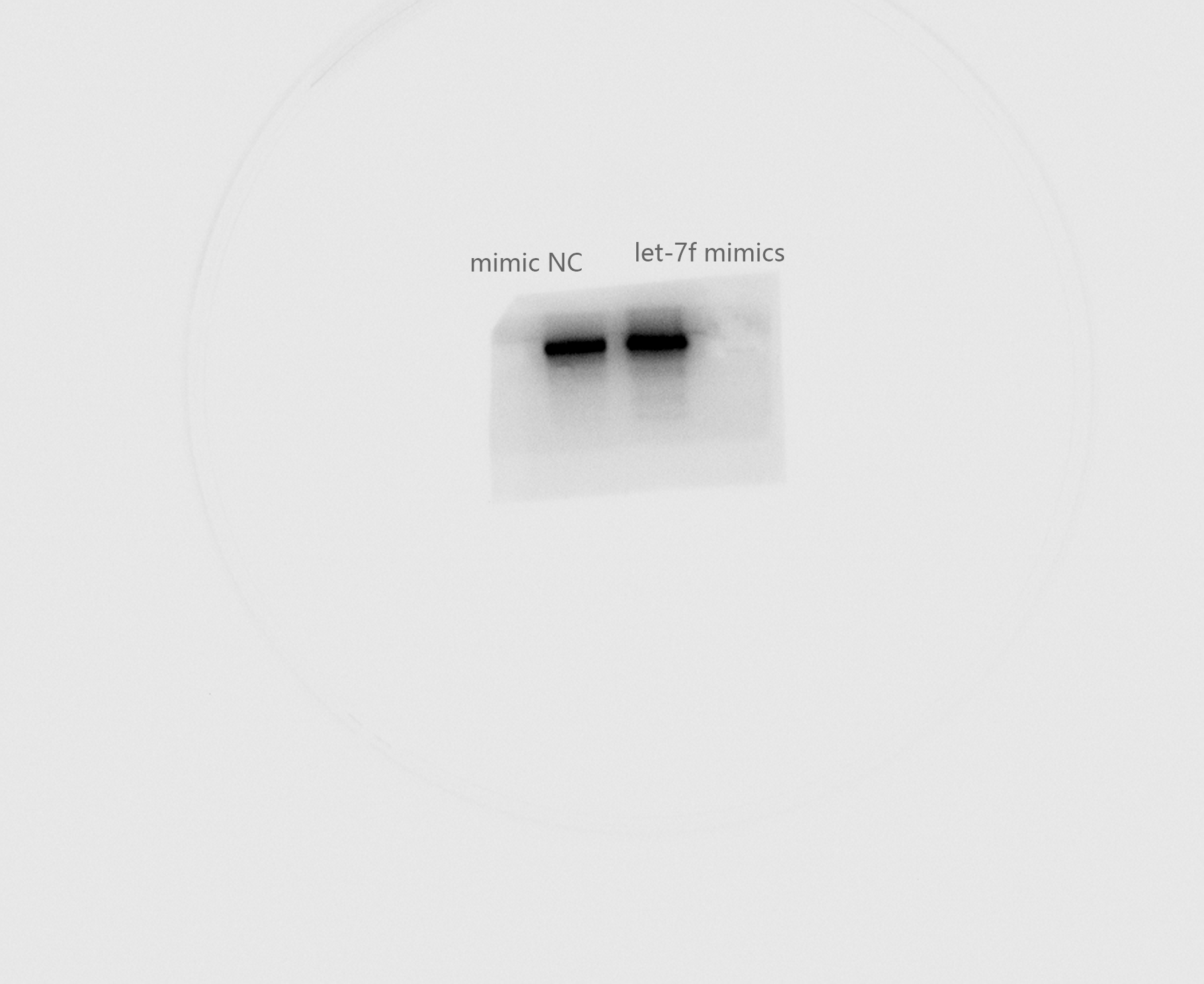

Supplement: Supplementary file 1 [file vetsci-11-00392-s001.zip › GAPDH- Repeat3.Tif]

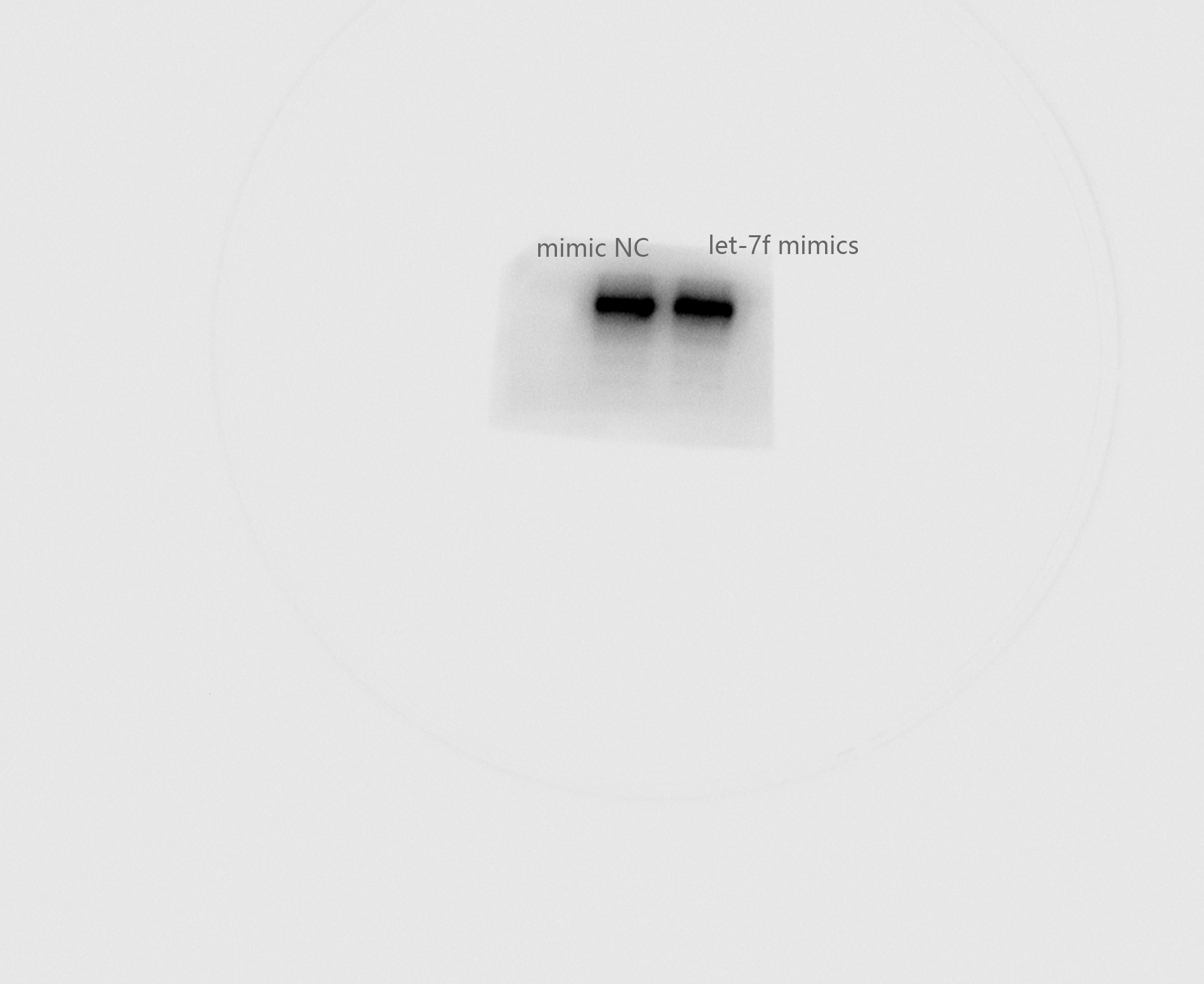

Supplement: Supplementary file 1 [file vetsci-11-00392-s001.zip › GAPDH-Repeat1.Tif]

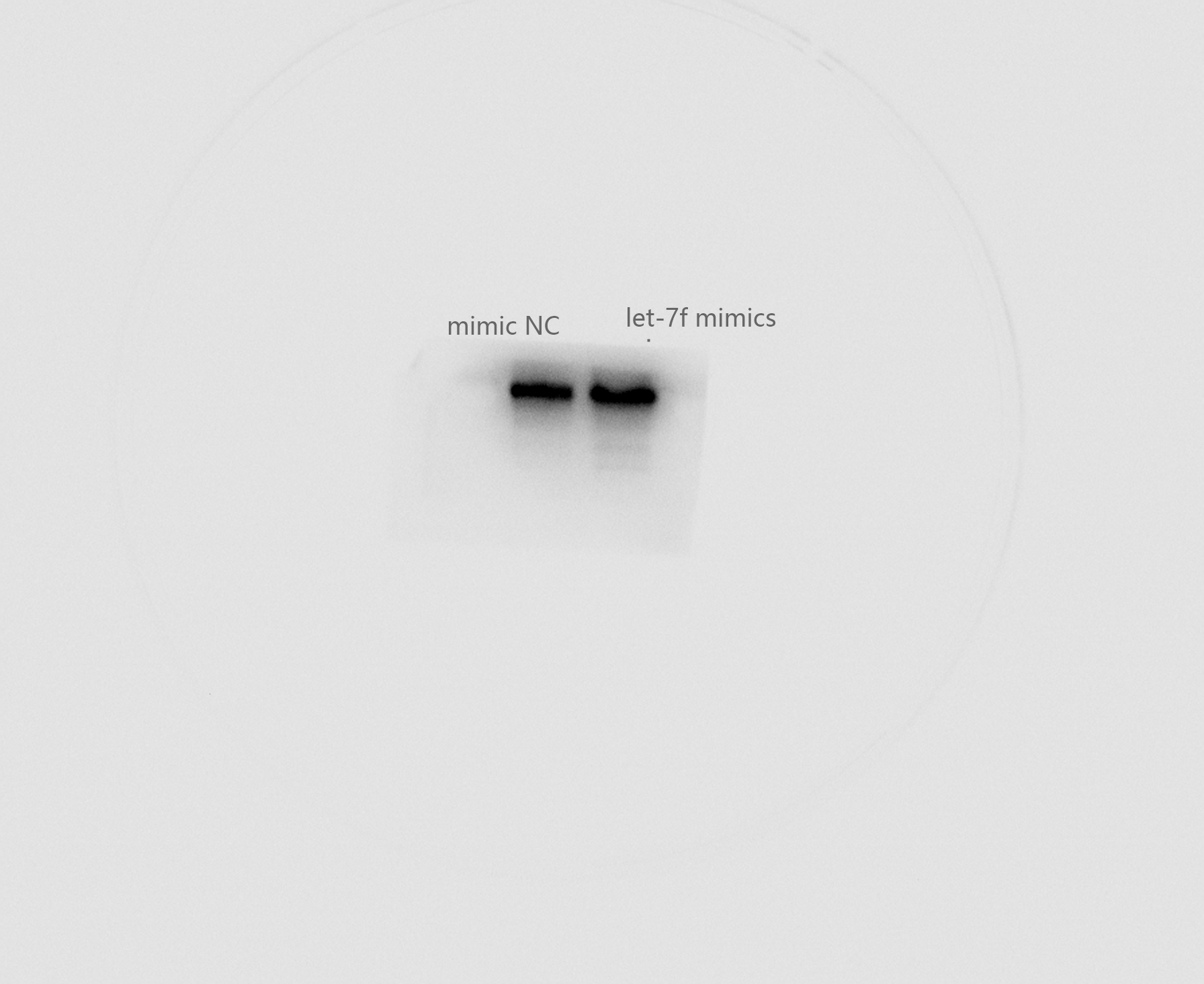

Supplement: Supplementary file 1 [file vetsci-11-00392-s001.zip › GAPDH-Repeat2.Tif]

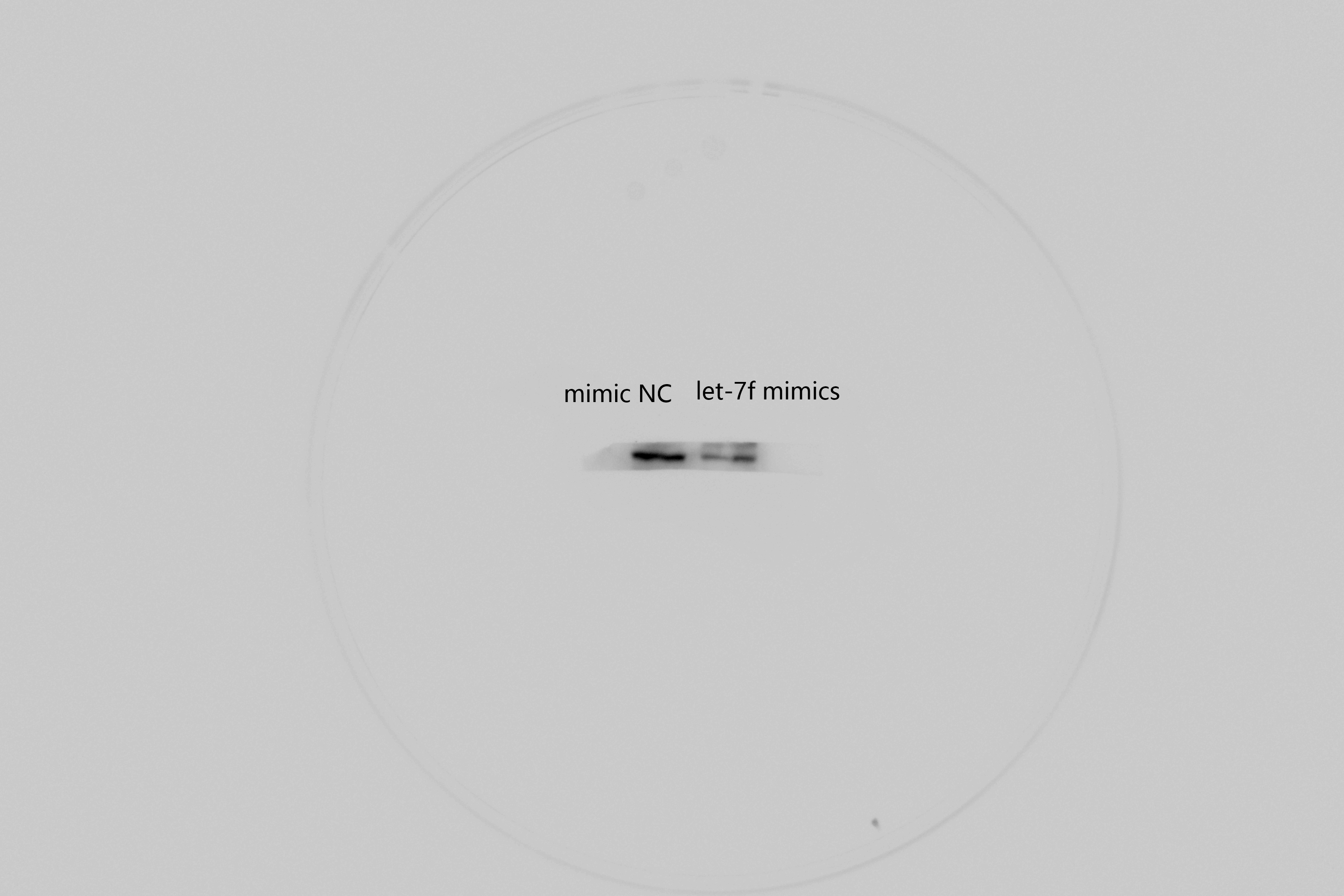

Supplement: Supplementary file 1 [file vetsci-11-00392-s001.zip › SREBP2-repeat1.tif]

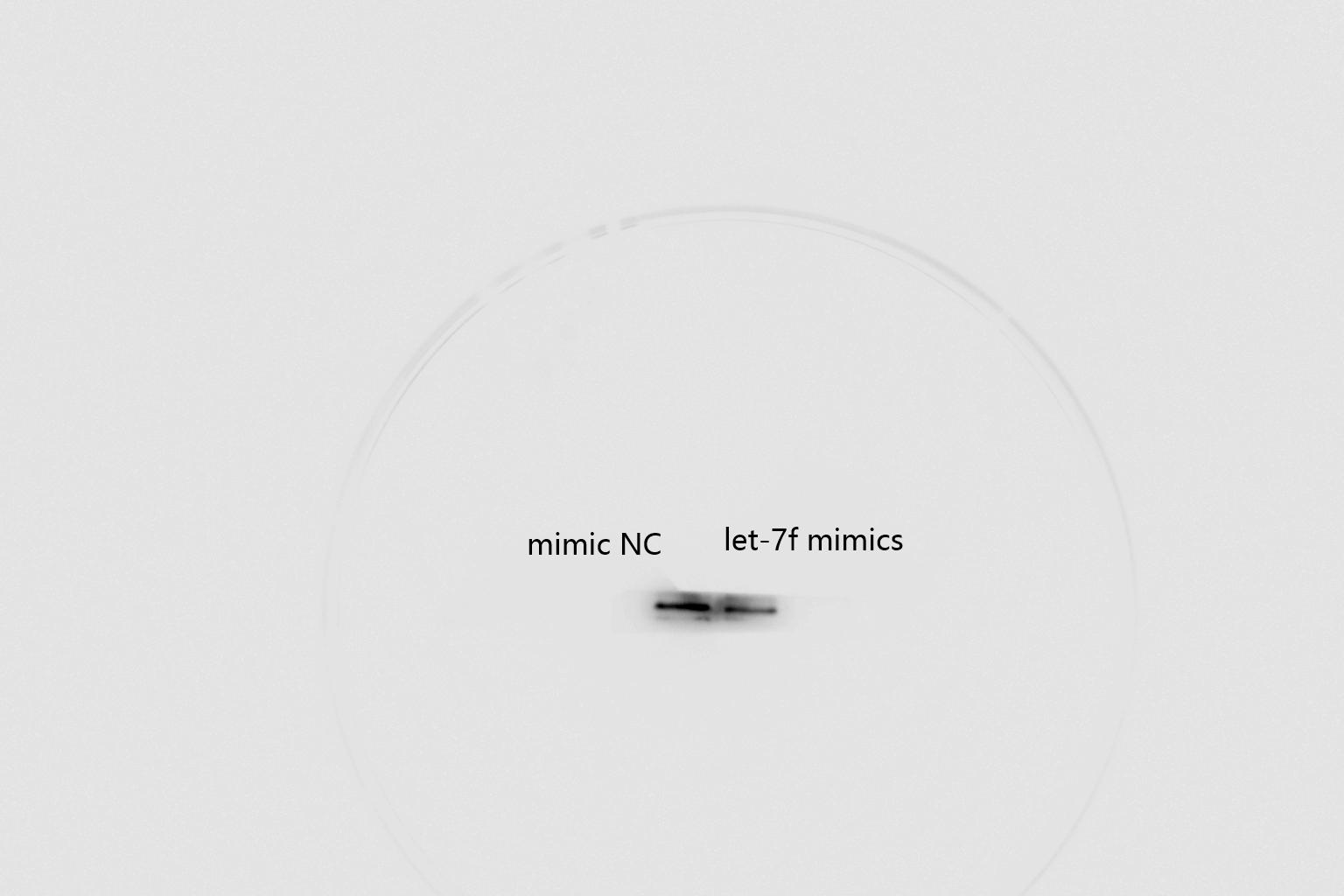

Supplement: Supplementary file 1 [file vetsci-11-00392-s001.zip › SREBP2-repeat2.tif]

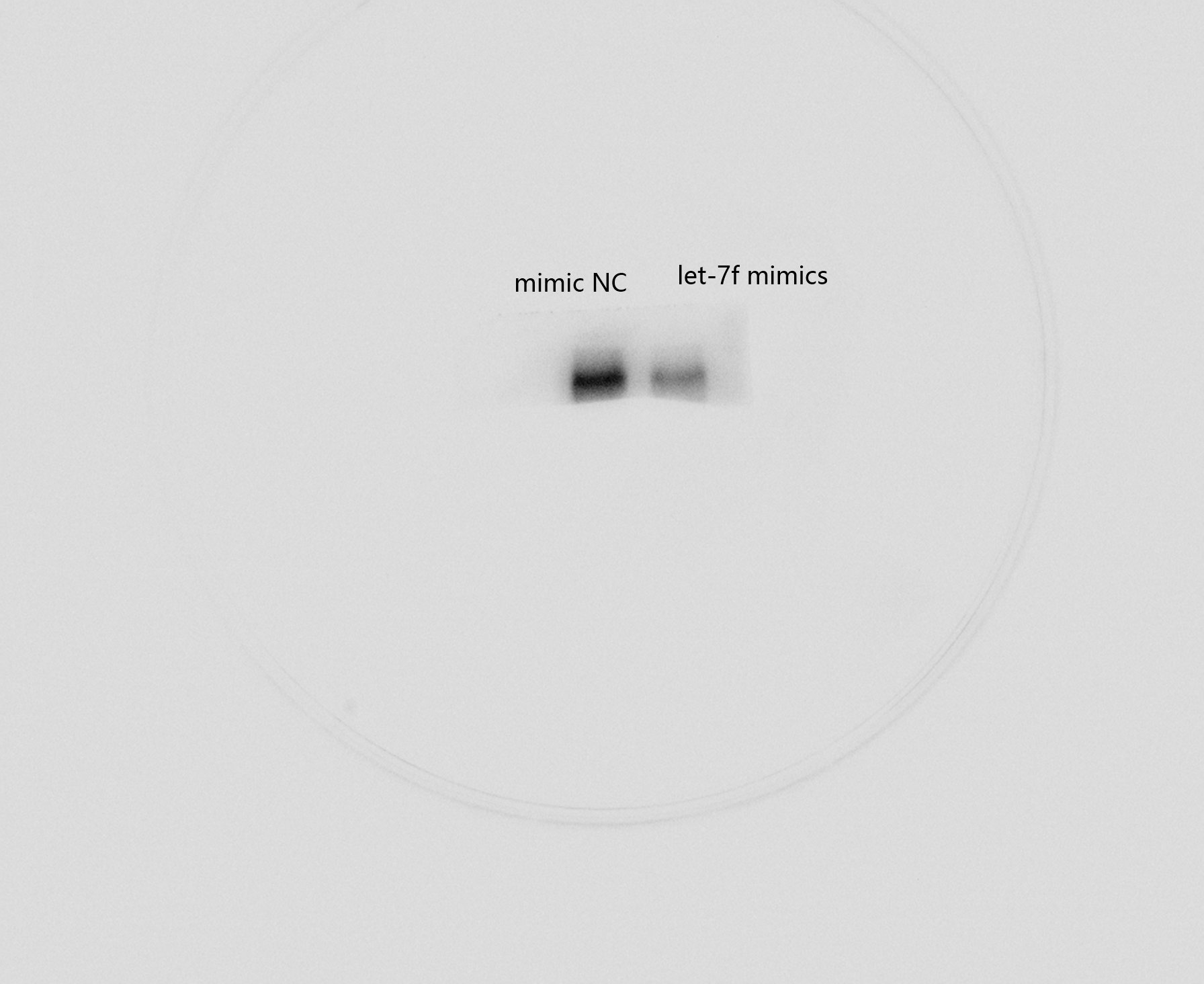

Supplement: Supplementary file 1 [file vetsci-11-00392-s001.zip › SREBP2-repeat3.Tif]
